# Nutritional Status and Obesity Paradox in Acute Coronary Syndrome Patients

**DOI:** 10.3390/jcdd13070314

**Published:** 2026-07-08

**Authors:** Alberto Cordero, Vicente Arrarte, Miriam Sandín, Óscar Moreno-Pérez, Emilio Flores

**Affiliations:** 1 Cardiology Department, Hospital General Universitario Dr. Balmis, 03010 Alicante, Spain; varrarte@gmail.com (V.A.); miriamsandinrollan@gmail.com (M.S.); 2 Instituto de Investigación Sanitaria y Biomédica de Alicante (ISABIAL), 03010 Alicante, Spain; omoreno@umh.es; 3 Departamento de Medicina, Universidad Miguel Hernández, 03202 Elche, Spain; 4 Centro de Investigación Biomédica en Red de Enfermedades Cardiovasculares (CIBERCV), 28029 Madrid, Spain; 5 Endocrinology Department, Hospital General Universitario Dr. Balmis, 03010 Alicante, Spain; 6 Clinical Laboratory Department, Hospital Universitario de San Juan, 03550 Alicante, Spain; flores_emi@gva.es

**Keywords:** nutritional status, obesity, acute coronary syndrome, mortality

## Abstract

The worse nutritional status of patients without obesity after an acute coronary syndrome (ACS) could explain their worse prognosis and the so-called obesity paradox. We performed a retrospective study of all consecutive patients admitted to a hospital for ACS between 2009 and 2020. Nutritional status was analyzed using the CONUT scale, and values > 4 were categorized as moderate-severe malnutrition. We included 2789 patients with a mean BMI of 28.0 (4.6) kg/m^2^ and 26.4% with obesity. The mean CONUT index was 2.4 (2.2), and 24.3% had moderate-severe malnutrition. Obese patients had a lower prevalence of moderate-severe malnutrition: 20.0% vs. 25.9% (*p* = 0.003). The median follow-up was 3 years. A significant interaction between BMI and the CONUT for mortality was verified, and both variables behaved in the opposite way in relation to risk. BMI was associated with lower all-cause (HR: 0.93, 95% CI 0.89–0.98) and cardiovascular (HR: 0.93, 95% CI 0.87–0.98) mortality risk only in patients with CONUT > 4; however, in patients without malnutrition, BMI obesity was not associated with increased mortality or major cardiovascular events. In conclusion, CONUT-defined nutritional-inflammatory status may partly contribute to the observed obesity paradox in ACS patients.

## 1. Introduction

Coronary artery disease and acute coronary syndromes (ACS) result from the interaction of classical cardiovascular risk factors. There is also an increasing number of non-classical determinants of atherosclerosis and cardiovascular vulnerability. Classical risk factors, such as age, male sex, hypertension, diabetes mellitus, dyslipidemia, smoking, and obesity, continue to be fundamental to cardiovascular risk assessment and prevention. However, recent evidence has emphasized that non-classical risk factors such as chronic inflammation, psychosocial stress, environmental exposure, socioeconomic status, frailty, sarcopenia, and nutritional status may also substantially influence the development and prognosis of cardiovascular disease [1]. This broader perspective is particularly relevant for patients with ACS, in whom the acute event often reveals a complex background of metabolic, inflammatory, and nutritional abnormalities that may not be fully captured by BMI or traditional risk scores.

Obesity and overweight increase the risk of developing established cardiovascular risk factors [2,3,4] and, also, the risk of major cardiovascular events [2,4,5]. In fact, most patients admitted for an ACS are overweight or obese [5,6]. Nonetheless, obesity is associated with a better prognosis after ACS, and this has been named the obesity paradox [2,7,8]. Several explanations have been proposed for this paradox, including differences in age, comorbidity burden, cardiorespiratory fitness, metabolic health, inflammatory status, and the limitations of BMI as a surrogate marker of body composition. Indeed, cardiorespiratory fitness [9] and metabolic status [10,11] have been identified as valuable predictors of mortality regardless of body mass index (BMI).

Malnutrition is common in patients with established cardiovascular disease [2,12] and can be present in nearly 40% of the patients admitted for an ACS [12,13]. Overweight or obesity is not incompatible with malnutrition, although this relationship has not been widely studied [2,14]. Therefore, the simultaneous assessment of BMI and nutritional status may help to clarify whether the apparent protective effect of higher BMI after ACS reflects true benefit or, alternatively, the adverse prognostic effect of malnutrition, frailty, and low body reserve among leaner patients. We hypothesized that the CONUT-defined nutritional-inflammatory status could provide a reliable argument for understanding the obesity paradox in patients with ACS.

## 2. Materials and Methods

We performed a retrospective study with all patients admitted for an ACS in a single center between 2009 and 2020. A total of 3339 were admitted, and albumin was available from 2789 (83.5%) of them, which constituted the cohort for the analyses. Nutritional status was assessed by the CONUT (Controlling nutritional status) scale, which is calculated using serum levels of albumin, lymphocytes, and total cholesterol [15]; patients with values > 4 were categorized as moderate-severe malnutrition. Patients with a BMI of 25–29.9 kg/m^2^ were characterized as overweight, and those with ≥30 kg/m^2^ as obese.

ACS was defined by the presence of typical clinical symptoms of chest pain and electrocardiographic changes indicative of myocardial ischemia/lesion and/or elevation of serum markers of myocardial damage [16]. Patients were classified as STEMI and NSTEACS according to the electrocardiographic findings. Risk factors, medical history, treatments, complementary tests, and principal diagnosis at discharge were registered from all patients by trained medical staff. Cholesterol remnant was calculated as total cholesterol minus low-density lipoprotein cholesterol (LDLc) minus high-density lipoprotein cholesterol (HDLc) [17]. Previous CAD was defined as having a clinical diagnosis of myocardial infarction, stable or unstable angina, or angina-driven coronary revascularization. Previous HF was codified if patients had at least one hospitalization with such a main diagnosis at a discharge-medical report, and those with typical signs and symptoms of HF that had a compatible imaging diagnosis (X-ray or echocardiogram). According to the 2019 ARC-HBR definition [18], patients were defined according to the ARC-HBR consensus if they met at least one major or two minor criteria. Glomerular filtration rate (GFR) was estimated using the CKD-EPI equation [19]. Comorbidities systematically collected in the medical history for this study were assessed by the Charlson index, adapted for patients with cardiovascular disease [20]; patients with a Charlson score > 4 qualified for high-comorbidity burden. According to current recommendations, optimal medical treatment (OMT) was codified when patients jointly received these four treatments: antiplatelets, statins, beta-blockers, and an angiotensin-converting enzyme inhibitor or angiotensin-receptor blocker [21].

The study’s primary endpoint was all-cause and cardiovascular mortality. The secondary endpoint was the time to the first major adverse cardiovascular event (MACE) that included ACS, unstable angina requiring hospitalization, unplanned revascularization, readmission for heart failure, stroke, or major bleeding.

The post-discharge follow-up of our registry has a well-established protocol based on phone calls, review of electronic medical reports, and institutional databases. The vital status was assured by phone calls in the absence of medical reports. The study was conducted following the STROBE (Strengthening the Reporting of Observational studies in Epidemiology) recommendations.

Quantitative variables are presented as mean (SD), and differences were assessed by ANOVA. Qualitative variables are presented as percentages, and differences were analyzed by Chi-square tests. The presence of interactions of covariates was tested in the survival analyses [22]. Survival analyses were performed by Cox-regression models, after verifying the proportional risk assumption by the Schoenfeld residuals test. Harrell’s C-statistic test was used to assess the model’s discrimination. Calibration was tested by the Gronnesby and Borgan test. Patients lost during follow-up were categorized as missing, as well as those who lacked any of the main variables for the analyses, although these were <5%. Prior to entry into regression models, the BMI values were expanded using fractional polynomials so as not to assume linearity of effect. Statistical difference was accepted at *p* < 0.05. All analyses were performed using STATA 14.3 (StataCorp. 2009. Stata Statistical Software: Release 14. College Station, TX, USA: StataCorp LP).

## 3. Results

As shown in Table 1, half of the cohort were overweight and one quarter had obesity. Significant differences were observed in most clinical features between patients with a BMI > 30 kg/m^2^ and the rest, especially in the prevalence of cardiovascular risk factors and previous cardiovascular disease, regardless of a significantly lower mean age. Patients with obesity presented less frequently as STEMI and had significantly lower GRACE scores; they also had a lower prevalence of high-bleeding-risk patients. As expected, patients with obesity had higher fasting glucose, glycated hemoglobin, triglycerides, and remnant cholesterol.

In-hospital mortality rate was 2.1% (52 patients), and a clear trend to a lower rate was observed for patients with lower BMI: 3.24% for patients with BMI < 25 kg/m^2^; 1.58% for patients with BMI 25–29.9 kg/m^2^; and 1.81% for patients with BMI > 30 kg/m^2^ (*p* = 0.05). In contrast, patients with CONUT scale > 4 had significantly higher in-hospital mortality rate: 5.13% vs. 1.49% (OR: 3.58, 95% CI 2.20–5.84; *p* < 0.01). Medical treatments recommended at the time of hospital discharge are presented in Table 2; no significant differences were observed, regardless of those related to diabetes, and optimal medical treatment was the same in the three categories of BMI.

**Figure 1:**
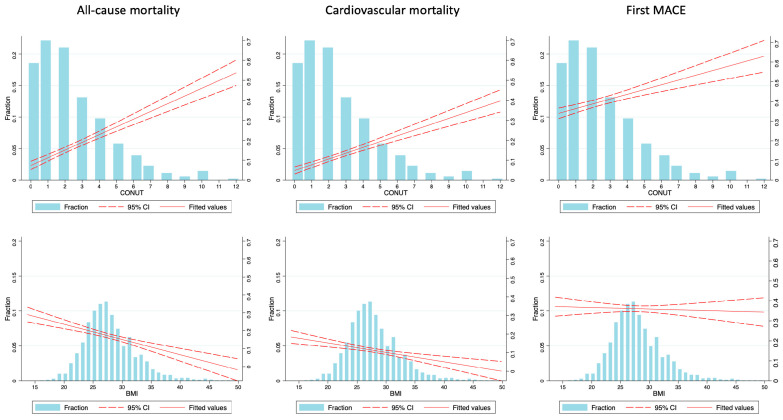
Distribution of body mass index (BMI) and CONUT scale and risk of all-cause mortality, cardiovascular mortality, or first major adverse cardiovascular event (MACE).

Post-discharge outcomes were available for 94% of the cohort, with a median follow-up of 1195 days (interquartile range 730–1581). All-cause mortality was 16.6% (416 patients), cardiovascular mortality 11.8% (295 patients), and 39.8% of the patients had at least one MACE. BMI and the CONUT scale had an opposite effect on all-cause and cardiovascular mortality, as well as on the first MACE (Figure 1).

**Figure 2:**
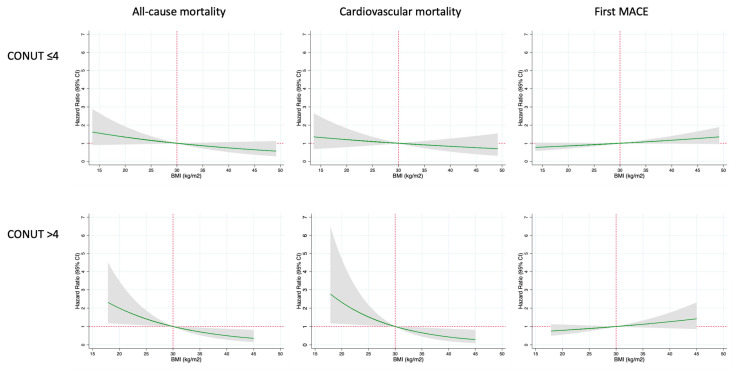
Adjusted risk of all-cause mortality, cardiovascular mortality, or first major adverse cardiovascular event (MACE) presented as hazard ratio (green line) and 95% CI (grey), associated with BMI (BMI) in patients with or without moderate-severe malnutrition (CONUT > 4).

A significant interaction between BMI and CONUT score was detected (*p* = 0.02) in the survival analyses and was included in further analyses. Multivariate analyses were adjusted by age, sex, diabetes, hypertension, previous coronary heart disease, heart failure or stroke, GRACE score, Charlson index, GFR, hemoglobin, revascularization, left ventricular ejection fraction, and medical therapies. The effect of BMI according to the absence or presence of CONUT-derived malnutrition state is presented in Figure 2. For patients with CONUT > 4, BMI was linearly associated with lower risk of all-cause (HR: 0.93, 95% CI 0.88–0.98; *p* = 0.01) and cardiovascular (HR: 0.92, 95% CI 0.86–0.98; *p* = 0.02) mortality and had not effect in the risk of MACE (HR: 1.02, 95% CI 0.99–1.05; *p* = 0.16). In contrast, in patients with CONUT < 4, BMI had no association with all-cause (HR: 0.97, 95% CI 0.94–1.00; *p* = 0.10) or cardiovascular (HR: 0.98, 95% CI 0.84–1.02; *p* = 0.37) mortality or MACE (HR: 1.02, 95% CI 1.00–1.03; *p* = 0.07).

## 4. Discussion

The analysis of this contemporary cohort of consecutive patients admitted for an ACS might clarify that the so-called obesity paradox could be explained by the effect of nutritional status. The significant interaction between the BMI and the nutritional status underscored how malnutrition modifies the risk related to BMI. Our results suggest that the higher mortality of patients with BMI < 25 kg/m^2^ with moderate-severe CONUT-defined nutritional-inflammatory status might provide a relevant argument for the lower mortality of patients with obesity and ACS, the so-called obesity paradox (graphical abstract). Since clinical characteristics of the cohort are similar to previous reports [13,15,23,24,25], we believe that our results might be representative and clinically useful.

The obesity paradox in ACS patients deserves careful consideration. Since all patients are included during an acute event, they all have overt cardiovascular disease, and taking lean subjects as the “reference group” might be biased by several reasons. First, subjects with a BMI < 25 kg/m^2^ that develop overt cardiovascular disease have a differential clinical profile, with higher rates of smoking and higher burden of comorbidities. Second, despite the lack of obesity, they have a higher prevalence of malnutrition, an independent predictor of poor outcomes [2,13,15]. Third, patients with BMI < 25 kg/m^2^ had the highest mean age and also presented more frequently as STEMI with a GRACE score > 140. We believe that these arguments underscore the peculiar characteristics of lean patients admitted for an ACS. In this line, a recent study with >60.000 subjects without obesity reported that 14% were metabolically unhealthy and 3% had embolic syndrome [10]. Similarly, weight loss has been associated with higher mortality rates in elderly patients [26], underlying the role of being lean as a marker of frailty. Similarly, a progressive increase in BMI is related to increased risk of acute coronary heart disease [27]. Our results are in concordance with all this evidence and highlight that patients with BMI < 30 kg/m^2^ had a higher Charlson index, reflecting a higher morbidity burden, and the highest rate of smoking. Thereafter, patients without obesity who develop an ACS have a high-risk clinical profile despite the lack of obesity.

The CONUT-defined nutritional-inflammatory status modified the long-term mortality risk and had an independent and strong prognostic value. The analysis of a large cohort of ACS, also from Spain, revealed that the highest prevalence of malnutrition was detected in patients with a BMI < 25 kg/m^2^ [13]. Investigators found discordant classification of nutritional status with different scores, but all were associated with a higher risk of mortality, reinfarction, or stroke [13]. Our results add evidence of an interaction between BMI and malnutrition and reinforce the need to evaluate nutritional status as an independent clinical feature in patients with cardiovascular disease. The reason why malnutrition increases mortality in these patients has not been fully described [2,13]. Low BMI usually involves low muscular mass or sarcopenia that also has an independent prognostic value [28]. We found that patients with moderate-severe malnutrition had more than 3-fold higher in-hospital mortality rates; the long-term follow-up also suggests that such a grade of malnutrition marks those patients with a BMI < 25 kg/m^2^ as having an increased risk of mortality. Interestingly, since there were no differences in revascularization rates or optimal medical treatment, and results were adjusted by the GRACE score, our results identified the independent prognostic value of BMI < 25 kg/m^2^ in patients with moderate-severe malnutrition.

The CONUT-defined nutritional-inflammatory status modified long-term mortality risk and had an independent and strong prognostic value. However, because CONUT includes albumin, lymphocytes, and total cholesterol, its prognostic value in ACS should not be attributed exclusively to malnutrition. Rather, the CONUT scale may integrate nutritional reserve, inflammatory activation, and metabolic status, all of which are biologically plausible determinants of prognosis after ACS [29,30].

In addition to medical treatments, all patients received recommendations related to smoking, exercise, and weight control according to current recommendations [31]. Since most patients were overweight or obese, most recommendations were probably directed to weight loss and to increase physical activity. Obesity and malnutrition are both modifiable risk factors, but much more effort is usually made on weight loss [2,23], and there is far less evidence related to malnutrition management [14]. In 2023, the European Society of Cardiology published, for the first time, a statement for the promotion of healthy nutrition in primary and secondary cardiovascular disease prevention [32]. Well-directed exercise programs can reverse the metabolic disorders related to obesity, but also improve strength and fitness that might have a deep impact on lean patients.

### Limitations

Our study has some limitations that should be addressed. We performed an observational, retrospective, and single-center study. Nutritional status was assessed only by the CONUT scale, which is one of the most accurate tools [13], but not the only one. Moreover, the absence of albumin may not be random, particularly in patients who are sicker, elderly, or those with shorter hospital stays or early death, which could have induced a selection bias. Overweight and obesity classifications were based on BMI, and other determinations could have helped to classify patients more precisely. Finally, the inclusion period was long, and, therefore, the use of concurrent treatments might have changed over the inclusion period. Despite these issues, as clinical features and long-term event incidence are similar to previous reports [13,15,23,24,25], we believe that the above-presented limitations might not have had a relevant impact on our results.

## 5. Conclusions

Nutritional status may partly contribute to the observed obesity paradox in ACS patients. BMI was associated with lower mortality rates only in patients with CONUT > 4, namely moderate-severe malnutrition. Integration of nutritional status might help to elucidate the effect of BMI on the prognosis of patients with cardiovascular disease and the so-called obesity paradox. Similarly, nutritional status should be assessed in all patients with established cardiovascular disease, as it is modifiable and has an independent prognostic value.

## Figures and Tables

**Table 1:** Clinical features of the cohort according to body mass index (BMI).

	Total	BMI < 25 kg/m^2^	BMI 25–29.9 kg/m^2^	BMI > 30 kg/m^2^	*p*
N	2789	718 (25.74%)	1334 (47.83%)	737 (26.42%)	
BMI (kg/m^2^)	28.0 (4.6)	23.1 (1.8)	27.3 (1.4)	33.8 (4.0)	<0.01
CONUT score	2.4 (2.2)	2.5 (2.2)	2.5 (2.4)	2.1 (1.9)	0.01 *
CONUT > 4	24.32%	25.93%	25.83%	20.03%	0.01
Age	68.30 (12.8)	69.1 (13.6)	69.3 (12.6)	65.7 (12.1)	<0.01 *
Women	24.97%	33.02%	21.45%	23.49%	<0.01
Diabetes mellitus	33.90%	27.62%	33.47%	40.81%	<0.01
Hypertension	65.22%	55.09%	67.36%	71.23%	<0.01
Dyslipidemia	51.95%	46.45%	54.07%	53.46%	<0.01
Current smokers	32.55%	38.15%	27.32%	36.62%	<0.01
Previous CHD	19.99%	14.81%	23.50%	18.67%	<0.01
Previous HF	2.27%	1.70%	2.24%	2.86%	0.37
Previous AF	10.02%	7.56%	10.71%	11.14%	0.05
Previous stroke	7.31%	8.64%	7.06%	6.48%	0.29
Peripheral arterial disease	7.99%	9.57%	7.72%	6.93%	0.19
Statin treatment before admission	31.98%	28.07%	33.46%	33.16%	<0.01
COPD	9.26%	9.26%	8.64%	10.39%	0.46
Previous cancer	5.92%	7.25%	5.73%	4.97%	0.20
STEMI	38.71%	43.06%	36.96%	37.65%	0.03
Charlson index	2.5 (2.8)	2.5 (4.0)	2.4 (2.2)	2.5 (2.1)	0.21
Charlson index > 4	23.21%	26.69%	20.40%	24.92%	<0.01
GRACE score	151.6 (45.3)	154.8 (45.4)	154.1 (46.0)	143.9 (43.0)	<0.01 *
GRACE score > 140	56.08%	58.80%	57.97%	50.00%	<0.01
High-bleeding risk	45.95%	52.01%	47.01%	38.10%	0.01
Revascularization	92.09%	89.51%	93.36%	92.32%	0.01
LVEF (SD)	54.4 (18.8)	54.0 (12.2)	54.5 (19.7)	54.7 (22.4)	0.68
Hemoglobin (mg/dL)	13.5 (5.1)	13.3 (5.1)	13.6 (5.6)	13.7 (4.3)	0.12
Fasting glucose (mg/dL)	124.9 (61.4)	121.6 (60.0)	122.4 (56.7)	132.3 (67.9)	<0.01 *
HbA1c (%)	6.4 (1.3)	6.1 (1.2)	6.4 (1.3)	6.6 (1.4)	<0.01
Total cholesterol (mg/dL)	161.9 (47.4)	162.4 (54.4)	160.1 (44.9)	164.7 (44.2)	0.07
LDL cholesterol (mg/dL)	94.1 (36.5)	93.8 (36.3)	93.1 (35.9)	96.1 (37.5)	0.10
HDL cholesterol (mg/dL)	41.7 (26.4)	45.2 (34.0)	41.7 (26.9)	38.3 (13.9)	<0.01
Triglycerides (mg/dL)	138.2 (90.5)	123.5 (90.2)	135.3 (81.9)	157.3 (101.4)	<0.01
Cholesterol remnant (mg/dL)	24.5 (26.8)	20.6 (38.6)	24.9 (20.5)	27.7 (21.8)	<0.01
Creatinine (mg/dL)	1.1 (0.5)	1.1 (0.6)	1.1 (0.5)	1.1 (0.6)	0.62
GFR (mL/min/1.72 m^2^)	73.3 (24.4)	73.5 (25.6)	72.0 (23.3)	75.5 (24.8)	<0.01 *
GFR < 60 mL/min/1.72 m^2^	28.08%	29.32%	30.01%	23.38%	<0.01
C-reactive protein	1.3 (0.3–3.8)	1.3 (0.4–4.6)	1.1 (0.3–3.7)	1.1 (0.4–3.2)	0.52

* for the comparison between the category of BMI > 30 kg/m^2^ and the other 2. COPD: chronic obstructive pulmonary disease; HDL: high-density lipoprotein cholesterol; GFR: glomerular filtration rate; LDL: low-density lipoprotein cholesterol; LVEF: left ventricular ejection fraction; STEMI: ST-elevation myocardial infarction.

**Table 2:** Treatments at discharge of the cohort according to body mass index (BMI) categories.

	Total	BMI < 25 kg/m^2^	BMI 25–29.9 kg/m^2^	BMI > 30 kg/m^2^	*p*
Aspirin	91.26%	90.27%	90.86%	92.94%	0.19
Clopidogrel	38.01%	37.32%	38.70%	37.42%	0.79
Ticagrelor	31.14%	33.33%	31.41%	28.53%	0.17
Prasugrel	18.54%	16.91%	18.12%	20.86%	0.17
Oral anticoagulation	12.38%	11.16%	11.81%	14.57%	0.12
ACEI/ARB	77.52%	74.16%	77.65%	80.52%	0.02
Beta-blockers	79.47%	79.27%	80.27%	78.22%	0.58
Diuretics	23.94%	21.05%	22.27%	29.75%	<0.01
Statins	92.85%	94.26%	93.06%	91.10%	0.09
Ezetimibe	9.88%	7.81%	10.33%	11.04%	0.12
Calcium channel blockers	14.72%	14.35%	13.46%	17.33%	0.08
Insulin	8.54%	6.86%	8.04%	11.04%	0.02
Oral antidiabetics	25.16%	16.59%	25.15%	33.44%	<0.01

ACEI: angiotensin-converting enzyme inhibitors; ARB: angiotensin receptor blockers.

## Data Availability

Data could be available under a reasonable request.

## Abbreviations

The following abbreviations are used in this manuscript:

ACS	Acute coronary syndrome
BMI	Body mass index
CONUT	Controlling nutritional status
LDLc	Low-density lipoprotein cholesterol
GFR	Glomerular filtration rate
